# Cyber–Physical Systems for High-Performance Machining of Difficult to Cut Materials in I5.0 Era—A Review

**DOI:** 10.3390/s24072324

**Published:** 2024-04-05

**Authors:** Hossein Gohari, Mahmoud Hassan, Bin Shi, Ahmad Sadek, Helmi Attia, Rachid M’Saoubi

**Affiliations:** 1 Department of Mechanical Engineering, McGill University, Montreal, QC H3A 0G4, Canada; hossein.goharibahabadi@mcgill.ca (H.G.); helmi.attia@mcgill.ca (H.A.); 2 Aerospace Manufacturing Technologies Center (AMTC), National Research Council Canada, Montreal, QC H3T 1J4, Canada; bin.shi@cnrc-nrc.gc.ca (B.S.); ahmad.sadek@cnrc-nrc.gc.ca (A.S.); 3 R&D Material and Technology Development, Seco Tools AB, SE-73782 Fagersta, Sweden; rachid.msaoubi@secotools.com

**Keywords:** process optimization, adaptive control, cyber–physical systems, industry 5.0, finite element analysis

## Abstract

The fifth Industrial revolution (I5.0) prioritizes resilience and sustainability, integrating cognitive cyber-physical systems and advanced technologies to enhance machining processes. Numerous research studies have been conducted to optimize machining operations by identifying and reducing sources of uncertainty and estimating the optimal cutting parameters. Virtual modeling and Tool Condition Monitoring (TCM) methodologies have been developed to assess the cutting states during machining processes. With a precise estimation of cutting states, the safety margin necessary to deal with uncertainties can be reduced, resulting in improved process productivity. This paper reviews the recent advances in high-performance machining systems, with a focus on cyber-physical models developed for the cutting operation of difficult-to-cut materials using cemented carbide tools. An overview of the literature and background on the advances in offline and online process optimization approaches are presented. Process optimization objectives such as tool life utilization, dynamic stability, enhanced productivity, improved machined part quality, reduced energy consumption, and carbon emissions are independently investigated for these offline and online optimization methods. Addressing the critical objectives and constraints prevalent in industrial applications, this paper explores the challenges and opportunities inherent to developing a robust cyber–physical optimization system.

## 1. Introduction

Manufacturing, which plays a vital role in the growth of the economy, has contributed to an estimated 24% of the U.S. Gross Domestic Product [1]. In addition, manufacturing has the largest economic multiplier of 3.05, i.e., each USD 1 of manufacturing output generates USD 3.05 in total economic activity [2]. As the final step in the manufacturing chain, machining claims up to 65% of all manufacturing processes, providing the required dimensional accuracy, surface quality, and other quality attributes. The cost associated with machining can exceed 65% of the product cost. This explains the adoption of advanced manufacturing technologies as a priority for government science and technology strategies [3]. The recognition of the potential conflict between economic growth and the protection of the environment, which is a natural capital (a source and a sink), has led to the need to put an end to economic growth in order to protect the environment, as presented in the “Limits to Growth” Report [4], published in 1972. Later, this view was changed, and it became clear that there is a need to revive economic growth globally, as outlined in the so-called Brundtland Report [5], which considered the environment and development as a single issue, introducing the concept of “sustainable development” (SD). The transformation of the economic growth–environment protection conflict into opportunities was one of the main drives for the industrial revolutions [6]. The fourth Industrial revolution (I4.0) represents a transformative paradigm in manufacturing, characterized by the integration of cutting-edge technologies to create smart, interconnected, and highly automated industrial systems. I4.0 aims to harness the power of cyber–physical systems, the Internet of Things (IoT), artificial intelligence, big data analytics, and advanced robotics to revolutionize the way products are designed, produced, and delivered. The seamless exchange of data across the entire value chain in I4.0 enables real-time decision making, predictive maintenance, and unparalleled efficiency gains, leading to intelligent, self-optimizing production processes. As recently as 2015, the fifth industrial revolution (I5.0) was introduced to overcome the shortfalls of its predecessor I4.0, which lacks key design and performance dimensions [7]. Although I4.0 and I5.0 share basic considerations of digital transformation, the customization of products, sustainable processes, and the creation of digital twins, the manufacturing paradigm I5.0 addresses other goals that include the possible customization of manufacturing processes, human–AI collaboration, and cognitive cyber–physical systems [8]. Additionally, I5.0 addresses the question of the sustainability strategy, side by side with the resilience strategy [6,7]. With this new vision, the I5.0 paradigm shift sets the ground for a framework that integrates high-performance manufacturing and sustainability [9].

The three pillars of I5.0 are resilience, sustainability, and human centricity. The resilience strategy requires interdisciplinary technologies that support creating cognitive cyber–physical systems (CCPS) to blend physical components and computing devices and to enable machine learning (ML) and artificial intelligence (AI)-based solutions to perform their functionality of the monitoring, control, and automation of physical processes, mimicking human-like cognitive processes, such as perception, reasoning, learning, decision making, communication, and collaboration [10]. For machining processes, other technologies that are needed include: (1) real-time process and tool condition monitoring (TCM); wear and sudden tool pre-failure detection using advanced AI and deep machine learning (DL) techniques [11], and wireless sensor-based smart tooling [12]. The data-driven training of the TCM system needs to be advanced to account for the variability in the signal features due to the physical phenomena that take place during the cutting of various classes of materials, e.g., metal matrix composites, biomaterials, and additively manufactured parts; (2) offline–online optimization and adaptive machining. This technology, which can easily be incorporated in a CCPS platform, was shown to improve productivity by up to 45%, and when integrated with a TCM, the production cost could be reduced by up to 25% [13]; and (3) physics-based constitutive models for anisotropic and graded materials that need to be developed and combined with artificial intelligence (AI) and swarm intelligent (SI) techniques to improve CCPS’s adaptability and scalability.

Process optimization is an essential element of the modern manufacturing industry and a key element of the industrial revolutions paradigm that can provide considerable improvements in terms of process productivity and product quality. Traditionally, machining parameters have been determined based on the experience of the machine tool operators, or through an experimental procedure. To mitigate the risk of catastrophic events, machining conditions are often chosen conservatively, albeit at the expense of limiting process productivity and increasing the environmental impact. In pursuit of more precise machining parameters, various process optimization methodologies have been developed. Improving process productivity is the main objective for machining process optimization in today’s competitive manufacturing industry. This can be achieved by reducing the cutting time and using the full potential of the cutting capacity of the machine tools. While the process productivity objective is commonly considered in optimizing roughing operations, the quality of the machined part is usually the main objective for the finishing operations [14]. The two main approaches to maximize process productivity are force/power-based optimization and material removal rate (MRR)-based optimization [15]. Force/power-based optimization provides a better performance than the MRR-based approach [16]. However, the implementation of the MRR-based approach is more feasible in industrial applications due to the complexity of the calculation of the uncut chip thickness and cutting forces, especially for free-form surfaces, in a force/power-based optimization approach [17].

Process optimization methodologies can be categorized into two main approaches: offline process optimization and online process optimization. In offline process optimization, a model is required to estimate one or multiple machining states based on the machining parameters. Analytical, numerical, and empirical approaches have been used to model the cutting operation. The output of the cutting model, directly or indirectly, is used to estimate machining objective functions, such as process productivity [18], machined part quality [19], production cost [20], tool life [21,22], chatter stability thresholds [23,24], energy consumption [25,26], and carbon emissions [27,28]. Online process optimization is defined as a numerical control process in which the machining parameters are regulated based on time-varying feedback [29]. The online optimization module is commonly implemented as a constrained optimization to regulate the cutting parameters, such as feedrate or spindle speed. This approach is designed to either limit or achieve consistency in key factors such as cutting force/power, tool wear rate, tool deflection, or their combined effects. The status of the constraints can be estimated directly from feedback signals such as the driving motor current, the cutting zone temperature, or acoustic emission (AE) signals. Alternatively, predictive information can be derived through analytical or statistical modeling approaches. An online optimization system can deal with the disturbances caused by the wide range of variations in the machining conditions. The implementation of an online control system can be highly beneficial to avoid deteriorating conditions such as chatter and excessive tool wear rate. In addition, the online optimization system mitigates fluctuations in cutting states, thereby reducing the likelihood of sudden tool breakage. The main drawbacks of implementing an online optimization system are the high cost of sensing, data acquisition, and processing equipment, as well as the difficulty in mounting sensors close to the cutting zone and the sensor sensitivity to harsh cutting environments [30]. Furthermore, achieving reliability and comprehensiveness in online process optimization systems remains a significant challenge.

**Figure 1:**
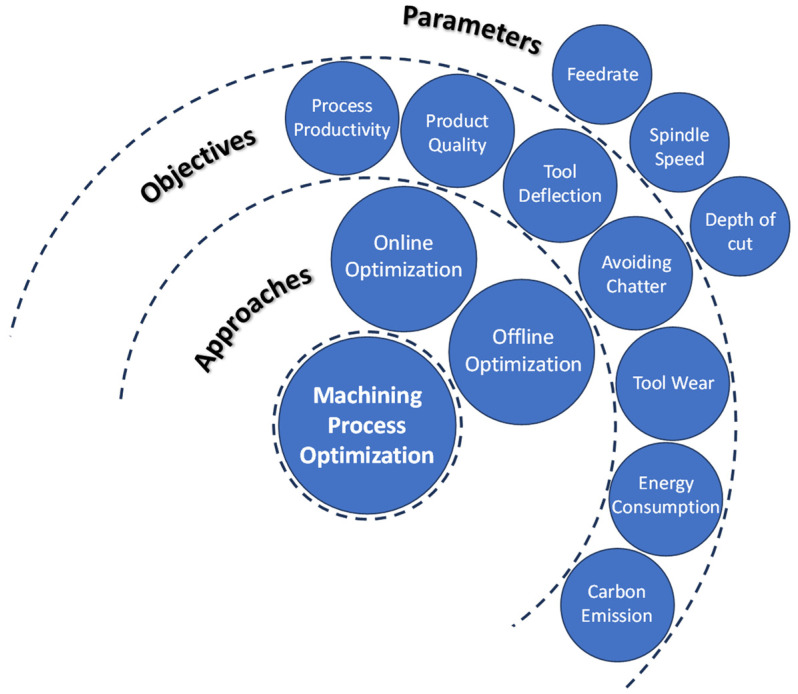
Machining process optimization, approaches, objectives, and optimization parameters.

The common machining parameters considered in machining process optimization are the feedrate [31,32], spindle speed [33,34], and depth of cut [35,36]. Feedrate is the most investigated machining parameter that can effectively optimize the machining time, part quality, energy consumption, and carbon emissions [31,37]. Feedrate optimization, also referred to as feedrate scheduling, is considered as the most effective machining optimization parameter, due to its flexibility in controlling the cutting states, such as cutting forces, tool deflection, tool wear, and surface error [30]. Spindle speed is widely considered in the optimization of workpiece surface quality, tool wear, and avoiding chatter vibration [38]. The depth of cut is the variable to be considered for optimizing machining thin-wall workpieces, where the maximum deflection of the workpiece is a crucial constraint [39]. Figure 1 shows a schematic representation of process optimization approaches, objectives, and parameters.

In both conventional and cognitive CPSs, a virtual model for the machining process is an essential element that allows for estimating the process states based on feedback signals from the physical system, and then modifying the process parameters [40]. The virtual machining system may consist of finite element models (FEM) of the cutting operation and crack propagation in the tool, tool/workpiece deflection models, surface roughness estimation models, engagement geometry models, and machine–tool–workpiece dynamic models. Developing numerical and analytical models for the machining process requires a deep understanding of the tool and workpiece materials’ mechanical, thermal, chemical, and microstructural properties, and interactions. The robustness and comprehensiveness of the virtual machining model are critical in the development of a CPS with a high level of intelligence and autonomy.

Currently, the huge investment in machine tools and other manufacturing equipment that are equipped with advanced embedded sensors for unmanned or closed-door machining creates the need for improved productivity to free these equipment’s capacity. The implementation of the CPS, within the context of smart manufacturing through digital transformation, has also gained considerable interest due to the ever-increasing demand for high quality and productivity at a low cost. In the high-performance machining of difficult-to-cut materials, an unoptimized machining process can lead to inefficient use of the machine tool capacity. Hybrid offline optimization and adaptive optimization control of the machining process in real time can result in the following benefits for industrial applicators and enhances their competitive position in the global market: (a) maximizing the production rate and reducing the machining time to free their equipment capacity, (b) lowering tool and production costs and eliminating/minimizing scrapped parts, (c) improved part quality by ensuring that cutting forces and temperatures are within pre-defined limits, and (d) optimizing cutting conditions and extending usable tool life. To achieve these goals, numerous works have been published in the literature to develop and integrate new models to predict the machining forces, temperature, and dynamic tool behavior to identify the corresponding optimization constraints. These models have been integrated with on-line process optimization models, using different schemes to maximize the process productivity throughout the toolpath. This paper explores the CPS framework within the high-performance machining domain, where computation and digitalization are integrated with physical processes like sensing and control. The primary objectives of this study involve the identification of crucial models for simulating and predicting cutting states, alongside investigating the methodologies, goals, and parameters essential for optimizing machining processes. Additionally, the paper investigates the incorporation of these models into both offline and online optimization procedures, revealing the challenges associated with aligning offline simulation and optimization with online monitoring and control in machining systems. Through a thorough review and analysis, this paper aims to illuminate the available solutions in the literature that deals with the complex dynamics of high-performance machining within CPS contexts, providing invaluable insights for advancing the understanding and application of the field. Special attention is given to the emerging fifth industrial revolution, I5.0. The specific objectives encompass a critical review of (1) the advancement of computational techniques for CPS, including fracture mechanics models, consideration of the tool microstructure, and thermal boundary conditions for sustainable machining processes, and (2) the optimization of the machining process in an adaptive control environment within the constraints of limiting chatter, tool wear, tool deflection, and environmental impact. Recent advancements in sensing and networking techniques are not covered in this review.

The paper is organized as follows: Section 2 introduces the framework for a CPS for machining systems. Section 3 describes the efficient multi-scale modeling techniques capable of being integrated into the CPS. This section highlights the important virtual models required to establish a digital twin for the machining system, such as material constitutive models, fracture models, thermal boundary conditions and heat transfer mechanisms, tool microstructure, and crack propagation models. These advancements unlock the potential for multi-scale physics-based predictions of tool wear. Section 4 provides a detailed examination of offline/online process optimization schemes, addressing the primary approaches, objectives, and crucial parameters. Finally, based on the studied elements, an envisioned cyber–physical system for high-performance machining is introduced.

## 2. Framework of Cyber–Physical Systems for Machining Processes

The concept of cyber–physical systems (CPS) was introduced in a workshop in 2006 as a new scientific foundation to develop novel engineering systems capable of rapid and reliable computation, communication, and control [41]. The framework of a CPS was further defined as an intelligent system incorporating monitoring, coordination, control, and integration tools in tightly interconnected computation and communication with the physical system [42]. The main challenges in the implementation of a CPS system for machining platforms are the difficulties in integrating the heterogeneous networks, systems, and devices, and processing massive data [43]. Recent advances in computer control systems, information technology (IT), and sensor manufacturing have provided a platform to develop a conventional or cognitive cyber–physical machine tool system.

**Figure 2:**
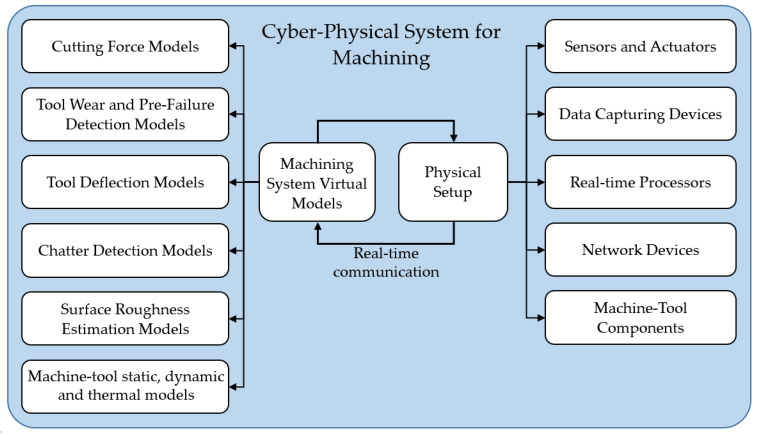
Components of a cyber–physical system for the machining process.

The first initiative on Cyber–Physical Machine Tool (CPMT) was introduced in a CIRP workshop in 2017, which categorized the system into four components: (1) CNC machine; (2) data acquisition; (3) digital twin for the machine tool; and (4) smart interfaces [40]. This concept requires a comprehensive virtual model for the machining process in a digital environment, along with real-time communication, measurement, and actuation in the physical environment. Virtual machining models consist of several analytical and numerical models related to each aspect of a cutting operation. Figure 2 is constructed to present the main components of a cyber–physical system for machining processes. Virtual models for machining states such as cutting force [44] and surface roughness [45,46] have been widely investigated. The main approach for estimating cutting forces is to use cutting coefficients extracted from orthogonal cutting tests for a specific set of tool–workpiece materials. A more accurate approach is to simulate the cutting conditions using a finite element modeling approach. This method can account for the effects of tool and workpiece microstructure evolution, as well as the dependence of workpiece flow stress on temperature, strain, and strain rate, among other factors. The cutting states such as forces and temperature are more accurately determined using the FE approach, considering the variations that may exist between the orthogonal tests with the actual cutting conditions. In addition, the detailed force and temperature distributions can be determined using a chip formation finite element simulation. Tool deflection models and chatter detection models are discussed in the offline/online process optimization section. To fully simulate the machining system, virtual models describing the static, dynamic, and thermal deformation of the machine tool structure should also be considered and integrated. Static errors in machining refer to inaccuracies in the position of the tool relative to the workpiece. These errors can arise from geometric inaccuracies in the machine and tool components and structural deformations caused by gravity and stationary forces [47]. Traditionally, direct measurement techniques such as using a laser interferometer or electronic levels were used to identify static errors. Indirect approaches such as the multi-line and body diagonal methods have been developed to more efficiently and accurately determine these errors [48]. There is no clear definition for dynamic errors so far, as pointed out in an extensive review presented in [47]. The general assumption is that dynamic errors are induced due to feed motions. This type of error could be significantly higher than static errors, especially during the high-speed machining of the sculptured surfaces. These induced errors are not only due to high feedrates, but also due to the acceleration/deceleration generated during the machining of high-curvature geometries and corners [49]. A common approach is to reduce these errors by interpolating the tool path in a way that limits acceleration and jerk throughout the path [50]. The work carried out by Attia and Kops introduced the effect of the machine tool structural joints on the machine thermal deformation. Later, this work was extended from offline predictions to real-time prediction and control to minimize the thermally induced errors that may reach 50% of the total machining errors [51,52,53].

DNN methods have been used to predict the chatter status more robustly during a machining process. Similarly, learning methods are implemented to predict the specific cutting forces [54]. In these approaches, training data are required as the input for the learning process, which can be acquired from experiments or numerical simulations. Due to the cost of the experiments and the variety of cutting conditions to be tested for training purposes, numerical models are the most feasible strategy. In the simulation of cutting processes, 2D and 3D orthogonal/oblique models of the cutting operation to simulate the chip formation are the prevailing approach, due to the simplifications that can be further correlated with a variety of cutting conditions [54,55]. In these simulations, the material constitutive model is the first and most important model required to predict the behavior of the material at very high temperatures, strains, and strain rates. Deriving a conclusive constitutive model to predict the thermo-mechanical behavior of the material is a challenging problem. The commonly used constitutive models in machining simulations are discussed in Section 3.1.

Advanced digital models capable of simulating the chip formation process and the dynamics of the machine tool are critical in developing a CPS. The modeling scope can be extended to encompass material handling, measurement, and inspection operations. Data collection throughout each manufacturing stage is facilitated by the incorporation of advanced tools, including Internet of Things (IoT) smart sensors, Radio Frequency Identification (RFID), and cloud storage [56]. Subsequently, these acquired data are employed for the generation of a Digital Twin (DT) for each manufacturing element, culminating in the establishment of a comprehensive cyber–physical systems (CPS) framework [57]. The data acquisition scope can be further extended to the in-service stage, with the gathered information being utilized for both product and process improvement [58]. Recently, artificial intelligence has been employed to improve the efficiency, accuracy, and comprehensiveness of virtual models for process simulation and product development. The application of the Chatty Factories concept, which leverages AI, Big Data, and an adaptive IoT/IT/OT security architecture in real-time processes, to machining applications has introduced a paradigm shift [58]. It dynamically enhances product design and the manufacturing process by incorporating insights from sensor-generated data. A study on chatty device use activity, utilizing clustering algorithms, highlighted the effectiveness of unsupervised machine learning in detecting unknown activities, which could practically initiate the “Chatty Factories” concept [59]. This not only optimizes production, but also allows for rapid adaptation to changing conditions.

## 3. Efficient Multi-Scale Modelling for Process Optimization

As research studies show, the proper selection of cutting parameters can reduce the effects of thermal stresses and deformations on the tool and workpiece by conveying more heat through the chip [60] and applying efficient cooling strategies. Implementing a CPS requires accurate and reliable virtual models to predict the cutting states and determine the optimum cutting parameters. Numerical simulation of a cutting process involves diverse physical models such as a material constitutive model, contact friction model, fracture criterion, and heat transfer equations. Finite element analysis (FEA) of a machining process is highly practical and beneficial for determining the proper selection of tool materials and provides information for optimizing the cutting parameters and developing models to predict tool wear and tool failure. Other numerical models for simulating the machining process include meshless and particle-based methods, the discrete element method, and the molecular dynamics (MD) simulation method. Meshless methods such as Smoothed-Particle Hydrodynamics (SPH) have been adopted as an alternative to the widely used FEM to handle large deformations in the workpiece [61,62]. Röthlin et al. [63] conducted high-resolution SPH simulations using scientific computing on a Graphics Processing Unit, GPU. The GPU acceleration of the SPH simulations enabled the inverse identification of constitutive model parameters within a numerical model of the cutting experiment [64]. Recently, using this numerical framework, computed process forces within the SPH model were used to train a machine learning model of orthogonal cutting [64] to improve the accuracy of force predictions over analytical and empirical models at similar low computational times. As another approach to overcome the challenges associated with large strains during cutting, which induce high deformation in the FE mesh, the Coupled Eulerian–Lagrangian (CEL) method was proposed by Ducobu et al. [65]. A review of the state of the art in the analytical and numerical modeling of conventional metal machining processes to predict chip formation, forces, temperatures, tool wear, residual stress, and microstructure is presented in a recent publication by Melkote et al. [66]. In this section, the material constitutive models, fracture model, and heat transfer model required for the chip formation simulation are discussed.

The contact friction model determines the frictional stresses acting on the rack and flack faces [67]. Friction models such as the constant shear friction factor for the whole cutting contact length and the constant shear friction factor for the sticking zone along with a constant friction coefficient for the sliding zone are the main simplified approaches that are implemented in machining FE simulations. Further sophisticated models to determine the variable shear friction factor and friction coefficient have been developed by establishing a relationship between the frictional stresses with the normal pressure at the contact faces [68]. Friction models have also been widely investigated and tested for the FE modeling of chip formation. A crucial model is the fracture criterion required to determine the failure condition for the material elements, considering the stresses, temperature, and strain acting on the element. These models were developed for different materials, where the model’s constants are first evaluated and then imported for the cutting simulation. The data needed for the process simulation are information on the thermal boundary conditions, which depend on the loading and cutting conditions. This information is particularly crucial if a coolant is present during the cutting process. Information on the thermal boundary conditions has also been widely investigated in the literature for different materials and cutting operations. The first challenge in research studies on machining is to predict the elastic–plastic behavior of the machined material alloy considering the strength variations at different temperatures and strain rates. The second challenge is to develop a finite element model (FEM) capable of predicting the cutting states considering the variations in the thermo-mechanical behavior of the machined material and the microstructure evolution.

Titanium-based alloys, e.g., Ti6Al4V, are preferred materials for aerospace, automotive, and biomedical applications due to their high strength-to-weight ratio, high corrosion resistance, their ability to maintain quality at high temperatures, and excellent biocompatibility [69]. However, these properties can significantly hinder their machinability, therefore, they can be defined as difficult to cut. This can be attributed to their high hardness, abrasion resistance, high strength at elevated temperatures, low thermal conductivity, and high chemical reactivity [70]. The generated high mechanical and thermal loads on the cutting edge can lead to different mechanisms of tool failure in terms of tool wear, chipping, or breakage. The machining of Ti-alloys is also characterized by the formation of segmental chips, which is governed by a ductile fracture mechanism, resulting in the cyclic variation of forces. The resulting vibration can limit the material removal rate and promote accelerated tool wear. The latter could have a negative influence on the surface integrity of the machined part [71]. The proper selection of the fracture criterion can significantly diminish the prediction errors in terms of the machining forces and machining-induced RS [71]. In the coming subsections, emphasis will be placed on some specific aspects that are particularly relevant to the modelling and simulation of machining difficult-to-cut materials, namely, the formulation of material constitutive laws, fracture models for predicting the effect of chip segmentation, modelling the material microstructure, and modelling the thermal boundary conditions and heat transfer process during cutting.

In this section, several critical aspects of multi-scale modelling for process optimization are covered. Attention is directed towards Material Constitutive Models (Section 3.1), Fracture Models in Chip Formation (Section 3.2), Thermal Boundary Conditions and Heat Transfer Models (Section 3.3), Microstructure Modelling (Section 3.4), and the Modelling of Tool Wear Considering the Tool Material Microstructure (Section 3.5). These components are examined to facilitate improved accuracy, predictive capabilities, and overall efficiency in machining processes. Additionally, the aim is to explore the interplay between various scales of modelling, enhancing the understanding of modelling complex machining phenomena and enabling more effective optimization strategies. This analysis is aimed at the development of robust and adaptable modelling techniques tailored to the intricacies of modern manufacturing environments.

### 3.1. Material Constitutive Models

Material constitutive models describe the elastic and plastic behavior of a material at different temperatures, strains, and strain rates. They are the essential part of finite element simulations of the cutting process, which highly affect the efficiency and accuracy of predicting the plastic deformation in the primary, secondary, and tertiary deformation zones. The main challenge in the simulation of a chip formation process is to determine the material behavior under severe deformation in a small region that occurs at high temperatures and high strain rates [72]. One of the widely used constitutive material models to predict the plastic behavior of the material under these conditions is the Johnson–Cook (J-C) model [73]. The main advantage of this model is its capability to estimate the low stress under large deformation. It is computationally more convenient for implementation. The general form of the J-C model is described as follows [74]:

(1) $$\begin{array}{c} \sigma =\left(A+B{\bar{\varepsilon }}^{n}\right)\left(1+C\ln\left(\frac{\dot{\varepsilon }}{{\dot{\varepsilon }}_{0}}\right)\right)\left(1-T^{*m}\right)\; \end{array}$$

where $T^{*m}=\frac{T-T_{ref}}{T_{melt}-T_{ref}}$, σ is the flow stress, $T^{*m}$ is the homologous temperature, $\dot{\varepsilon }$ is the strain rate, *A* is the yield stress at the reference temperature and reference strain rate, B is the strain hardening coefficient, *C* is the strain rate hardening coefficient, *n* is the strain hardening exponent, and *m* is the thermal softening exponent. Typical values of the J-C model parameters for Ti6Al4V reported in literature are listed in Table 1. Each term is interpreted as a thermo-mechanical flow characteristic. The first term, $\left(A+B{\bar{\varepsilon }}^{n}\right)$, depicts the strain-hardening phenomenon, while the second term, $\left(1+C\ln\left(\dot{\varepsilon }/{\dot{\varepsilon }}_{0}\right)\right)$, describes the strain rate effect and the third term, $\left(1-T^{*m}\right)$, represents the thermal-softening effect. Optimization approaches such as PSO and fireworks have been suggested to more accurately determine the coefficients based on a set of experimental data [75]. Recently, it has been suggested to estimate the J-C model parameters through neural network models to eliminate the necessity for extensive experiments and characterize the coefficients based on different cutting conditions, which improves the accuracy and efficiency of utilizing the J-C model in finite element simulations [76]. In-depth reviews of the methods for identifying the coefficients of the material constitutive models for the cutting processes are presented in [77,78]. In these studies, a method is developed based on the information obtained from the distributed primary zone deformations (DPZD), the quasi-static indentation (QSI) tests, and the orthogonal cutting tests at room temperature and a high temperature, which eliminates the errors that commonly occur in the simulation of severe plastic deformation.

The next popular constitutive model is the modified Zerilli–Armstrong model, which predicts the flow stress behavior of the material at certain temperatures, strains, and strain rates. Dislocation mechanisms are the main deriving factors to estimate the plastic behavior of the material [80]. The equation of the modified Zerilli–Armstrong model is shown in Equation (2):

(2) $$\begin{array}{c} \;\sigma =\left(C_{1}+C_{2}\varepsilon^{n}\right)\exp\left\{-\left(C_{3}+C_{4}\varepsilon \right)T^{*}+(C_{5}+C_{6}T^{*})ln{\dot{\varepsilon }}^{*}\right\}\; \end{array}$$

where σ is the flow stress, $T^{*}=T-T_{ref}$, *T* is the temperature, $T_{ref}$ is the reference temperature, $\dot{\varepsilon }$ is the strain rate, and $C_{1}$ to $C_{6}$ and *n* are the material constants. Table 2 shows an example of these material constant values for Ti6Al4V. Metaheuristic optimization such as the Genetic algorithm has been suggested to reduce the inaccuracies in the determination of the constants [83].

Another important constitutive model is the Voyiadjis–Abed model, in which the thermomechanical properties of a material are determined based on a physical-based approach rather than an empirical curve fitting method [84]. This method was originally developed for pure FCC, BCC, and HCP materials to describe their plastic behavior based on their internal microstructural features [85]. The Voyiadjis–Abed equation is represented in Equation (3).

(3) $$\;\sigma =C_{1}+C_{2}\varepsilon_{p}^{C3}+C_{4}{\left(1-{\left(-C_{5}Tln\frac{{\dot{\varepsilon }}_{p}}{{\dot{\varepsilon }}_{p}^{*Y}}\right)}^{\frac{1}{q_{1}}}\right)}^{\frac{1}{q_{2}}}+C_{6}\varepsilon_{p}^{C_{7}}{\left(1-{\left(-C_{5}Tln\frac{{\dot{\varepsilon }}_{p}}{{\dot{\varepsilon }}_{p}^{*H}}\right)}^{\frac{1}{q_{1}}}\right)}^{\frac{1}{q_{2}}}$$

where σ is the flow stress, $C_{1}$ to $C_{7}$ are the coefficients related to the internal microstructure characteristics of the material, $q_{1}$ and $q_{2}$ are the constant exponents, and ${\dot{\varepsilon }}_{p}^{*Y}$ and ${\dot{\varepsilon }}_{p}^{*H}$ are the determined reference strain rates for the yielding and hardening mechanisms. Table 3 lists typical values of the Voyiadjis–Abed model constants for Ti6Al4V.

**Figure 3:**
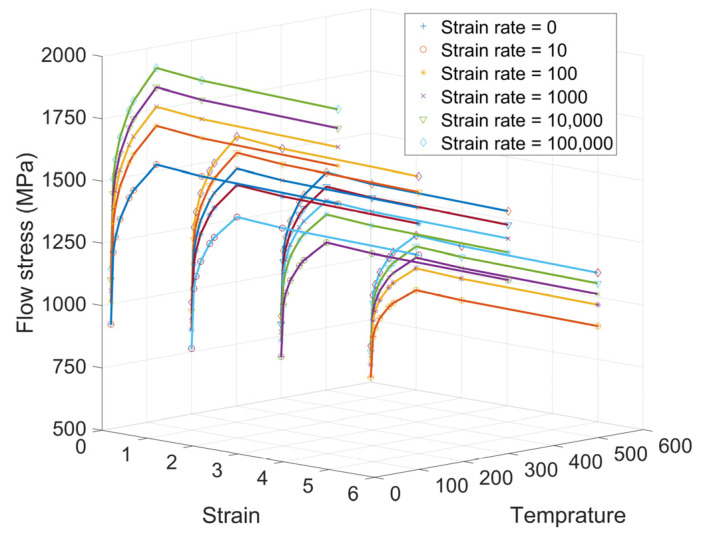
Tabulated flow stress data for Ti6Al4V at four different temperatures and six different strain rates extracted from Deform^®^ software (https://www.deform.com/, accessed on 30 March 2019) [87].

To estimate the flow stress at different stresses, strains, strain rates, and temperatures, a tabulated flow stress model based on experiments can be constructed. Deform^®^ is a major commercial finite element software developed specifically to simulate manufacturing processes. It employs a tabulated flow stress model as a default approach for the constitutive material model to evaluate the flow stresses. The general format of tabulated flow stress can be represented as $\bar{\sigma }=\bar{\sigma }\left(\bar{\varepsilon },\dot{\varepsilon },T\right)$, where $\bar{\sigma }$ is the equivalent flow stress, $\bar{\varepsilon }$ is the equivalent strain, $\dot{\varepsilon }$ is the strain rate, and *T* is the temperature. A linear weighted average interpolation scheme is used to determine the flow stress based on a set of tabulated data points [86]. A representation of tabulated flow stress data for the Ti-alloy Ti6Al4V is shown in Figure 3. This model has more accuracy and compatibility with experimental data and is considered as the benchmark for comparing the accuracy of the other constitutive models developed for Ti6Al4V [86]. A study on the finite element simulation of the cutting process by Liu et al. [86] showed that the Johnson–Cook model developed by Leseur [82] for Ti6Al4V has a better compatibility with the Deform^®^ software tabulated data (published in 2019) in comparison with the other mentioned approaches.

There have been several attempts to combine machine learning and artificial intelligence in the finite element simulation of machining processes [88,89]. These approaches can increase the efficiency, reliability, and accuracy of the simulation and can be utilized for a wider range of cutting conditions. They have the flexibility to be interconnected with the physical system and can be used to establish a decision-making core to enhance the information perception and control of the manufacturing system.

### 3.2. Fracture Model in Chip Formation

As experimentally observed, cyclic chips, also known as serrated or segmented chips, are the dominant chip morphology in the machining of titanium alloys in the conventional cutting speed ranges. This phenomenon is explained through two main theories known as the ductile fracture mechanic and the adiabatic shear theory [90]. The ductile fracture mechanic suggests that serrated chips form as a consequence of crack initiations on the chip’s free surface, and they spread to the tooltip periodically. The primary shear zone is weakened by periodic cracks, resulting in chip segmentation. The adiabatic shear theory assumes that serrated chips are created by periodic thermoplastic shear instability inside the primary shear zone [91].

The common fracture model incorporated into modelling chip segmentation in machining Ti6Al4V is Cockroft and Latham’s criterion [92]. This model can be represented as follows:

(4) $$\int_{0}^{{\bar{\varepsilon }}_{f}}\sigma_{max}d\bar{\varepsilon }=C\;$$

In which ${\bar{\varepsilon }}_{f}$ is the equivalent plastic strain at which fracture happens, $\sigma_{max}$ is the maximum principal stress, and *C* is the material damage value. The integral value is calculated for each element in a finite element simulation. If the integral reaches the material constant, the solver considers the element as a damaged element and deletes it. The reported values for the material constant for Ti6Al4V are in the range from 100 to 400 MPa, which can be calibrated through an iterative procedure by comparing the predicted and measured chip geometries and the principal cutting force [81]. As shown experimentally, the cutting speed has a considerable effect on the chip geometry, resulting in rising the frequency of chip segmentation when the cutting speed is increased [81,93]. The corresponding material constant in Cockroft and Latham’s criterion increases in the simulation of a high-speed machining condition [81].

### 3.3. Thermal Boundary Conditions and Heat Transfer Models

**Figure 4:**
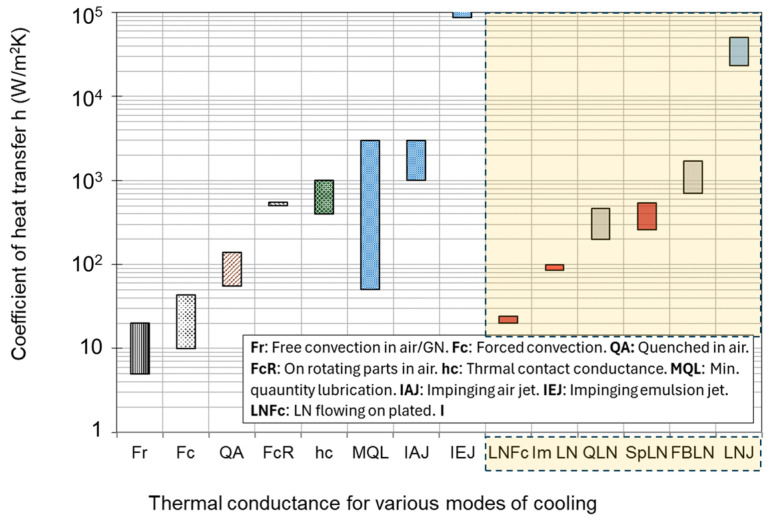
Typical values of the coefficient of heat transfer (CHT) in machining under different cooling regimes [99].

The main sources of heat generation in a cutting operation are the dissipation of plastic deformations to heat in the primary and secondary deformation zones, and the friction-induced heat at the tool–chip interface [94,95]. As studies have shown, the generated heat due to the friction at the contact region between the tool and chip is considerably smaller than the heat generated from the plastic deformation in the shear zones. To improve the machinability of the Ti6Al4V alloy and other difficult-to-cut materials, several cooling approaches have been introduced, such as minimum quantity lubrication (MQL), the cryogenic cooling method, and high-pressure coolant (HPC) [96,97]. The cryogenic method is a more advanced approach for cooling the cutting zone, especially for materials with a low thermal conductivity [98]. However, the application of the cryogenic cooling method for Ti6Al4V alloys increases the material hardness, mechanical loads, and tool wear rate [70,96]. HCP is recognized as a low-cost and maintenance approach for the cooling process in the machining of Ti alloys [70]. Cooling information is required for determining the boundary conditions for the designed cutting operation. Figure 4 shows the typical ranges of the coefficient of heat transfer (CHT) in machining under different cooling regimes [99]. Table 4 represents the heat transfer information required in the finite element simulation of machining operations.

### 3.4. Microstructure Modeling

Microstructure modelling of the tool and workpiece material could significantly improve the accuracy of the numerical simulation of a cutting operation, especially for multi-phase materials. Multi-phase materials like cemented carbides are highly affected by internal microstructure features such as grain size, shape and distribution, constituent phases, and interfacial properties [102,103]. WC/Co cemented carbide, also referred to as a hard metal, is a crucial alloy employed in a variety of industrial tools, such as cutting inserts, drilling bits, and dies, due to its outstanding combination of hardness and toughness [102]. The two contributing phases of this alloy, known as tungsten carbide (WC) and Cobalt (Co), have different mechanical properties. The WC phase is considered to be the brittle phase, which contributes to the hardness of the alloy and behaves elastically under loading conditions, while the Co phase, referred to as the binder, is the ductile phase, which contributes to the toughness of the alloy and represents its elastic–plastic behavior under loading conditions [104].

**Figure 5:**
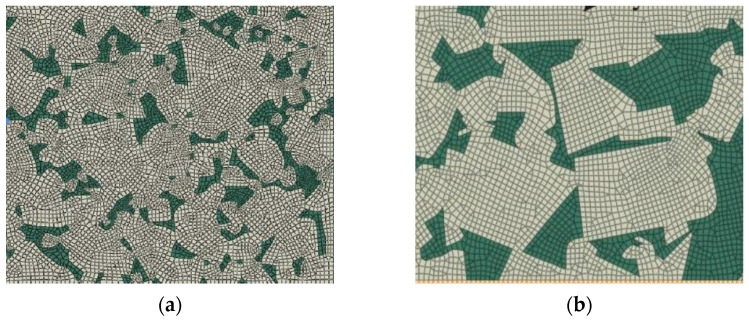
Examples of FE mesh extracted from the real microstructure of WC/Co. (**a**) WC-10 Co %wt [109] and (**b**) WC-20 Co %wt [110].

To model a heterogeneous material in continuum mechanics, a representative volume element (RVE) is usually developed, which represents the microstructural characteristics of the composite material. One of the dominant approaches for developing an RVE for a polycrystalline microstructure is to replicate the real microstructure properties of the material captured by a scanning electron microscope (SEM) or electron backscatter diffraction (EBSD) microscope [105]. Software such as object-oriented finite element in 3D (OOF3D) [106] and Materials Image Processing and Automated Reconstruction MIPAR™ [107] has been developed to analyze and segment the images captured from the microstructure of the material. Examples of constructed real microstructure meshes for the WC/Co microstructure are illustrated in Figure 5. Machine learning algorithms have been implemented in the image processing of microstructure images to classify the pixels based on the material phases. Pulse-coupled neural networks (PCNN) have been found as a robust method for segmenting microstructure images for generating FEM meshes [108].

**Figure 6:**
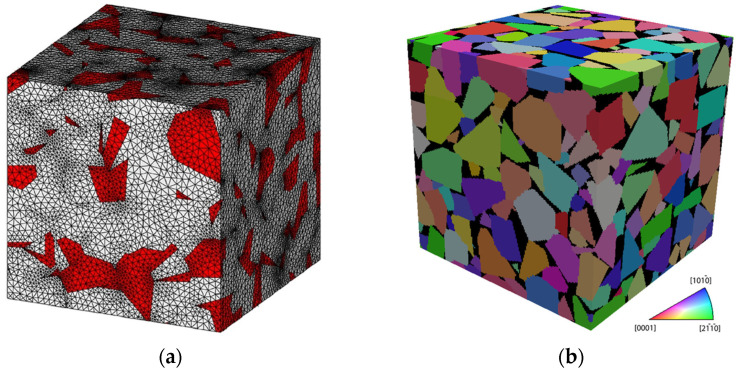
Examples of synthetic microstructures generated for WC/Co. (**a**) WC-15 Co %wt [104] and (**b**) WC-10 Co %wt [103].

The second general approach in constructing a multi-phase material microstructure is to synthetically generate the microstructure using various statistical and numerical methods, such as Voronoi tessellations [111], a synthetic grain structure builder (DREAM.3D) [112], Monte Carlo [113], and CCBuilder [103]. The determined microstructure from these approaches can be converted into finite element meshes. Figure 6 shows two synthetic microstructures generated for a WC/Co material based on the (a) Voronoi tessellation method and (b) CCBuilder software. Information such as grain size, grain shape, and neighbor distributions is needed to generate a synthetic microstructure. This information can be extracted from a 2D image of the real microstructure.

### 3.5. Modelling of Tool Wear Considering the Tool Material Microstructure

**Figure 7:**
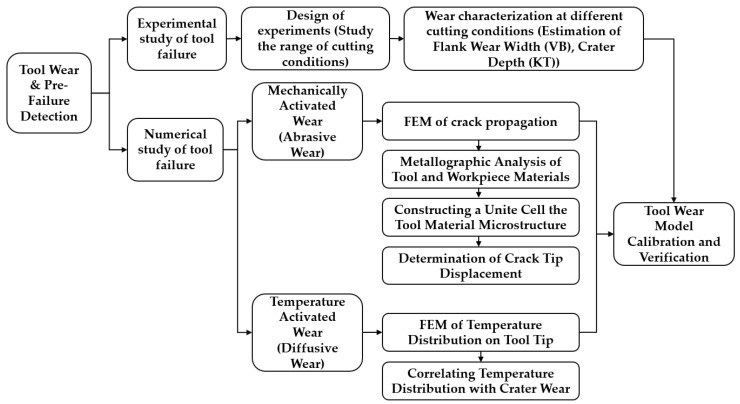
Tool wear and pre-failure detection module.

The friction between the cutting tool and workpiece during a machining process gradually wears and deforms the cutting edge. The development of wear mechanisms on the two main sides of the cutting edge has a direct relationship with the cutting time. Five mechanisms are known that contribute to the development of flank wear and crater wear, which are: abrasion, attrition, adhesion, diffusion, and oxidation [2]. Diffusion and oxidation are categorized as temperature-activated wear mechanisms, while abrasion, attrition, and adhesion are the mechanically activated wear phenomena [114]. The occurrence of each type of these mechanisms in a machining operation is dependent on the cutting tool material, workpiece material, and cutting conditions. Diffusive wear occurs during sliding contact between the tool and the workpiece, which facilitates chemical bonding between the tool materials with the workpiece material. To develop a virtual model for tool wear and pre-failure detection, each of the wear mechanisms is required to be modelled. Figure 7 depicts a proposed approach to predicting the tool wear and pre-failure detection module. Both mechanically and temperature-activated wear approaches can be considered in developing a virtual model for a cutting process. The intensity of each wear mechanism varies by changing the tool and workpiece material and cutting conditions. As experimentally observed in [114], mechanically activated wear occurs due to subsurface crack propagation in the cobalt binder of WC/Co tools.

Temperature-activated wear is numerically or empirically simulated, which is critical in the machining of difficult-to-cut materials such as titanium alloys [115]. Recently, Malakizadi et al. proposed a new approach to predicting the thermally activated dissolution-diffusion wear of carbide tools [116], considering the effect of the alloying elements on the solubility of tool materials in highly alloyed workpiece materials. A calibrated thermodynamic model is combined with the FE model of machining process, which considers the thermal constriction resistance at the tool–chip interface. The approach can efficiently simulate the nonlinear tool wear process without resorting to costly iterative FE simulations.

Mechanically activated wear can be simulated based on the finite element simulation of crack propagation in the tool material [117]. In order to model the temperature-activated wear mechanism, it is important to determine the temperature distribution at the interfaces of the tool–chip and tool–workpiece. The heat and stress distributions on the tool rake face can be determined using a finite element model of chip formation. This information can then be used to calculate the rate of progression in the size of the crater.

It has been found that cracks can extend through both the brittle and ductile phases of WC/Co alloys. The fracture starts in the carbide phase based on a brittle mechanism, and after the creation of a multi-ligament zone (MLZ), it continues through the Co binder in a ductile manner [118]. The basic assumption for crack propagation under a cyclic load is defined based on the slider motion between two surfaces [119]. In traditional approaches, crack propagation is analyzed locally based on the influence of the loading conditions defined away from the cracks. This approach is more accurate in predicting the ductile fracture properties for isotropic and homogenous materials [120]. An important factor in crack propagation analysis is the material resistance to the crack extension, which can be varied at different crack lengths and different materials, especially for materials with elastic–plastic behavior and anisotropic microstructure properties. The two main strategies for simulating crack propagation in cemented carbides are the simulation of small crack progress based on crack tip displacement (CTD) analysis and mesoscale crack propagation simulation based on continuum damage mechanics (CDM). Crack propagation analysis based on the CTD criterion is mainly applied for one cycle stress loading conditions [121].

It has been observed that the crack growth process consists of three distinguished stages on the plot of crack extension per cycle (da/dN) versus the logarithmic scale of changes in the stress intensity factor (∆K). An empirical model is proposed to create a relationship between ∆K and da/dN at the intermediate region where the curve is linear, as shown in the following equation [122]:

(5) $$\frac{da}{dN}=C{\left(∆K\right)}^{n}\;$$

where a is the crack length, *N* is the number of cycles, ∆K is the range of the stress intensity factor, and *C* and *n* are the material constants.

It was revealed that the crack growth at the first stage occurs due to the extension of small cracks, which cannot be accurately estimated by linear elastic–plastic fracture mechanics (LEFM) [114]. The size of small cracks was found to be in the range of the grain size in monolithic materials and the inter-particle spacing for the composite materials. This phenomenon shows that the real mechanism of crack propagation at small scales is different from that of longer cracks [114]. It was found experimentally that the crack propagation rate (CPR) of small cracks changes with the variations in the crystallographic orientation of the grains and the adjacent cracks [123], and small cracks propagate along the primary slip system direction [124]. Shear decohesion of the slip bands near the crack tip is recognized as the crack propagation mechanism for small cracks, where these cracks propagate along the direction of maximum shear stress [125]. The proposed model to determine the propagation rate when there is a mixed mode of loading (normal and shear stresses) is represented in the following equation [114]:

(6) $$\frac{da}{dN}=A{\left(\Delta CTD\right)}^{n}$$

where $\Delta \vec{CTD}=\left|∆\delta_{P}+∆\delta_{s}\right|$ is the crack tip displacement determined from the primary and secondary slip components calculated at the tip of the crack, *A* is an empirical constant, and *n* is the exponent of the fatigue crack growth equation. The analytical calculation of ΔCTD can be extremely complicated. Further developments revealed that the ΔCTD can be determined from finite element modelling to calculate the resultant CTD from the crack tip sliding displacement ∆CTSD and the crack tip opening displacement ∆CTO, as represented in the following equation [126]:

(7) $$∆CTD=\sqrt{{∆CTSD}^{2}+{∆CTOD}^{2}}$$

where CTSD is defined as the relative displacement of two nodes at the upper and lower crack surface in the tangential direction to the crack plane and CTOD is the relative displacement of two nodes that are attached to the upper and lower crack surface in the normal direction to the crack plane. CTD is a more accurate crack-tip-characterizing parameter when the plastic strain energy component is considerably high at the tip of the crack in comparison with the elastic strain energy component [120].

Mesoscale crack propagation based on CDM is applicable for a high number of cycle loads and can be used to trace the crack extension [127]. Crack propagation modelling provides information for predicting and detecting tool failure. There have been studies correlating the crack propagation rate with the AE emission signal, which can be used in the pre-failure detection of cutting tools [128]. An autonomous and comprehensive approach can be developed for industrial applications that can deal with all the possible uncertainties and disturbances using the introduced hybrid approaches for the modelling and optimization of cutting processes.

**Figure 8:**
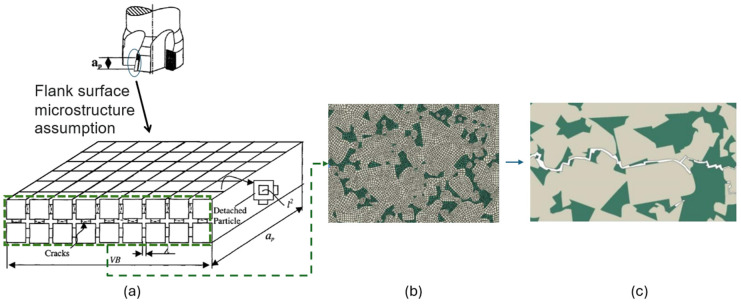
(**a**) Schematic representation of the wear model in milling using cemented carbide tools [121,126], (**b**) real microstructure modelling of the tool material [109], and (**c**) crack propagation model of under cyclic load [110].

An essential aspect of a cyber–physical machining system is to predict the tool life and adjust the cutting conditions to utilize the full potential of the tool while avoiding excessive wear rates. A wear model developed based on the finite element of crack propagation in the tool microstructure can determine the wear status more accurately, as presented in [121,126]. In this study, the flank face of the tool was partitioned based on the average size of the WC grains of the tool material, as shown in Figure 8a. The normal and tangential stresses acting on the flank land were determined based on the cutting conditions (depth of cut, feed rate, and cutting speed) and the tool geometry (rake and clearance angles). The rate of grain detachment was evaluated based on the applied stresses and the number of grains engaged on the flank face. Figure 8b shows the result of a tool wear model developed based on a simulation of crack propagation in a tool microstructure. The figure displays the wear results at two different cutting speeds (2000 and 6000 m/min) and feedrates (mm/tooth). The model could accurately determine the tool wear during the second stage of wear, where the wear behavior is linear with respect to the cutting length. From this information, the changes in the size of the flank wear land can be determined more accurately under different cutting conditions. This model can be combined with a TCM system to account for the uncertainties involved in a cutting operation.

## 4. Offline/Online Process Optimization for Cyber–Physical Systems

Machining process optimization considers one or multiple objectives of process productivity, energy consumption, part quality, production time, and cost. Production time and cost exhibit a close interrelation and can be regarded as subgoals in enhancing process performance through fully utilizing the capacity of the machine–tool setup. Cutting force and power, as well as tool and workpiece deflections, have direct relationships and can be considered in optimizing both process productivity and final part quality. Material removal rate (MRR) is another cutting state indicator that is directly correlated with the cutting forces and commonly considered in improving machining productivity. Tool wear, energy consumption, and sustainability are the recently invested machining objectives that are essential in creating a green production that satisfies the I4.0 and I5.0 manufacturing paradigms. Tool wear directly affects energy consumption, cutting force, and product quality [129,130]. In all cases, identifying the physical limits of the machining platform and the tool–workpiece engagements are crucial for implementing a process optimization module.

This section focuses on strategies to improve productivity, economics, part quality, and sustainability within the CPS framework, encompassing offline, online, and hybrid models. Various aspects crucial to achieving an optimal performance in machining operations are addressed in terms of productivity, economics, part quality and process sustainability. Through these sections and subsections, a comprehensive understanding of process optimization through cyber–physical systems in machining environments is sought to be provided.

### 4.1. Productivity and Economics

This subsection is dedicated to productivity and economics within the context of offline/online process optimization for cyber–physical systems. Various methodologies aimed at enhancing efficiency and cost-effectiveness in machining operations are discussed. Offline, online, and hybrid strategies such as Tool Wear Monitoring (Section 4.1.1) and Process Parameters Adaptive Control (Section 4.1.2) are examined. Through the examination of these approaches, insights into optimizing production processes to achieve a higher productivity and improved economic performance are aimed to be provided.

#### 4.1.1. Tool Wear Monitoring and Control

An inaccurate selection of cutting parameters could cause damage to the cutting tool, resulting in premature tool failure, increased tooling costs, and part damage. Poor control of tool wear in machining leads to out-of-tolerance parts and increased machine down-time, which, indirectly, may account for 30% of the total machining cost [131]. The cutting speed is the most crucial parameter affecting the tool life and surface quality in machining hard-to-cut materials [132]. Generally, the goal is to employ higher cutting speeds to achieve a higher material removal rate and reduce the lead time. However, this leads to a significant increase in tool wear. The state of tool wear has a significant impact on machining optimization objectives such as energy consumption and product quality [129]. Tool wear mechanisms can be attributed to mechanical [126], thermal [133], and chemical [134] aspects, making the wear phenomenon a complex modelling problem. Traditionally, direct tool wear evaluation techniques such as using a microscope, CCD (charged-coupled device) camera, or laser beam have been used to assess the status of tool wear. Due to access limitations that exist during machining, such as a lack of proper illumination and the presence of cutting fluid, indirect tool wear measurement techniques were developed to continuously estimate the tool wear in an online monitoring system [135,136]. Several machining process signals, such as cutting forces, vibration, temperature, AE, displacements, and spindle power, were utilized to estimate the tool wear state. Among these signals, cutting forces, vibrations, and AE have been more frequently used to estimate tool wear state [137]. The relationship between machining parameters, acquired signals, and the tool wear state is highly nonlinear, and developing analytical formulations may be inaccurate [138]. Therefore, data-driven approaches have been widely adopted in the literature.

**Figure 9:**
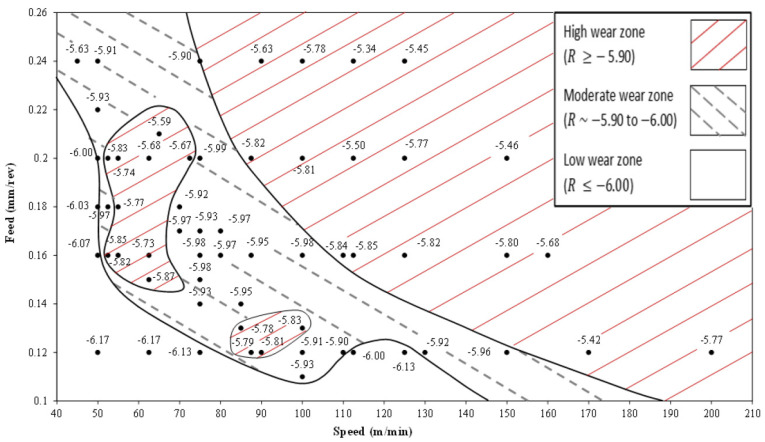
Tool wear map generated for machining Ti6Al4V [141].

Developing a wear map for the selected machine–tool–material setup is the primary technique used in the offline optimization approach to determine the optimal cutting parameters [139,140]. Figure 9 illustrates a wear map developed for machining titanium alloys using an uncoated tool. Using this map, one can choose the feedrate and cutting speed in areas where the tool wear rate is minimum. The tool wear rate is commonly defined as the logarithmic value of the fraction of flank wear over the cutting length [141].

A decision-making module in an intelligent manufacturing system estimates the proper time for the tool change that maximizes tool utilization and avoids any possible damage to the workpiece [142]. Prasad et al. [143] developed an adaptive control machining system for a numerical turning operation, in which the process is constrained based on a set of predefined thresholds. The developed self-tuning system adjusts the cutting parameters (cutting speed, feedrate, and depth of cut) to maintain the flank wear and tool deflection under specific limits that are defined for a certain workpiece. Employing the design of experiments and statistical analysis to determine the optimal cutting parameters is the dominant approach for minimizing tool wear [144]. Methods such as Taguchi signal/noise-based optimization [145,146], ANOVA and response surface methodology (RSM)-based optimization [147,148], particle swarm optimization (PSO), and its combination with adaptive neuro-fuzzy inference systems (ANFISs) [129] are commonly developed to minimize tool wear. Table 5 presents a summary of the developed optimization systems to control or limit the tool wear.

In another recent study, a combined TCM and adaptive control (AC) system was developed for the drilling process to improve the machining efficiency and reduce the machining time and cost [153,154]. As can be seen in Table 5, the dominant approach to modelling tool wear is to use statistical models that are based on experimental procedures. The main available offline/online models were developed for drilling operations, where the cutting engagement is simpler than that in a milling operation. A CPS equipped with a TCM should be capable of detecting changes in tool conditions, while remaining insensitive to fluctuations in cutting conditions and AC environments, as presented in [151,153]. Furthermore, it should have a high level of decision-making certainty, requiring minimal learning efforts, and should be capable of performing signal processing and decision making within a proper time frame [154]. A possible solution is to combine a hybrid analytical–numerical model, such as the one presented in [152], with a TCM system and an AC module. This approach can improve the comprehensiveness of the CPS. Further improvements are required to develop reliable and accurate tool wear monitoring and a control strategy specifically for milling operations.

#### 4.1.2. Process Parameters Adaptive Control

Cutting forces originate from several mechanical and thermal interactions that occur at the tool–chip and tool–machined surface interfaces. The resistance of workpiece material to plastic deformations in the primary and secondary deformation zones and the friction between the tool and the workpiece material on the tool rake and flank faces are the main sources of generating the cutting forces. A machining process usually contains a variety of engagements between the cutting tool and workpiece, which causes fluctuations in cutting forces and induces vibrations. The inability to select optimum feedrates to avoid high cutting forces is a common problem, particularly during roughing operations, where the main goal is to maximize the material removal rate (MRR). The instantaneous cutting force and power can be considered as comprehensive indicators of the cutting state, as they have direct relationships with the MRR, cutting temperature [155], tool/workpiece deflection, tool failure, tool chipping, and tool wear [30]. In offline force-based process optimization modules, the MRR is estimated by comparing the cutter locations (CL) with the stock geometry and the cutting parameters extracted from the NC code [38]. Based on the determined cutting engagements, cutting forces are estimated through mechanistic force models [156]. The main challenge in calculating the cutting forces is to keep track of the cutting engagements to determine the instantaneous cut geometry [30]. The machined surface topography and texture can be improved, as well through cutting force control, by manipulating the feedrate along the tool path to maintain constant cutting forces [30,157].

**Figure 10:**
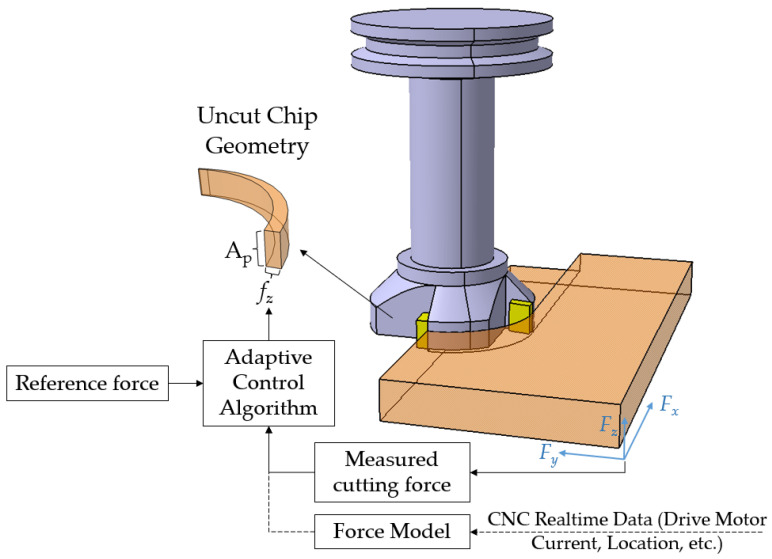
Schematic representation of adaptive cutting force control in a milling process.

Figure 10 represents a schematic diagram of an adaptive control system with cutting force constraints. An online process optimizer with a cutting force constraint can be implemented to maintain the measured or estimated cutting forces close to the level of reference forces. The latter can be determined based on a virtual model of the cutting operation considering the machine tool setup characteristics and the limits of the maximum tool deflection, cutting temperature, and tool wear rate [29]. Alternatively, force and/or power sensors can be utilized. Since the maximum cutting force has a direct linear relationship with the chip thickness, the online optimization system can control the cutting forces by manipulating the uncut chip thickness, as presented in Figure 10. The main challenge in implementing an online optimization system in low-volume–high-variety discrete manufacturing is the prolonged learning effort and lead-time. Model-based controllers were the first developed online process optimization systems to regulate the CNC motions, considering the external limits and variations in cutting conditions [158]. The main difficulties in implementing these systems to deal with complex and harsh cutting conditions are the complex computations required in real time and the dependency of the system on the accuracy and reliability of the external sensors [159,160]. For example, the force measurement sensors are sensitive to shock and rapid fluctuations of the cutting parameters, resulting in a high noise-to-signal ratio of the acquired signals [161].

The feasibility of implementing online optimization systems for industrial applications has improved over the last decade as the cost and quality of available sensors have decreased. In addition, mathematical, numerical, and statistical models that accurately assess and monitor machining operations have recently evolved. These advancements have contributed to the development of robust and accurate online optimization systems. A summary of the developed optimization systems to control force/power and MRR is presented in Table 6, which shows that offline process optimization is the primary approach to optimizing cutting operations. In a recent study performed by the authors (published in 2023) [13], an offline/online optimization approach was developed for milling operations, in which offline optimization of the cutting forces was implemented based on a limit for the tool deflection, along with online power optimization to reduce the cutting time and avoid excessive tool wear and high thermal stresses at the tool–chip interface.

The other important observation from the recent developments is the emergence of machine learning methods for modelling the machining process, which could provide more comprehensive and robust results to deal with the uncertainties in cutting operations. Hybrid approaches of conventional and AI-based methodologies for AC systems have been recently adopted to estimate, maintain, or constrain cutting forces during machining using a combination of neural networks, fuzzy logic, and metaheuristic optimization [162]. The combination of statistical modelling methodologies along with heuristic optimization has been the main trend in optimizing the cutting forces to achieve the maximum MRR [129,163]. This approach requires a series of experiments at various levels of each cutting parameter. The outcome of these experiments is then used to develop a model that can be used to find the optimal cutting parameters. Despite the accuracy of such developed approaches, they are time-consuming and do not fit modern dynamic industrial facilities. More research is needed to assess the performance of these methods for industrial applications.

**Table 6:** Developed machining process optimization based on cutting force and MRR.

Approach	Objective	Methods	Feedback	Machining Process
Offline	Power-constrained optimization [31]	An iterative optimization approach constrained with the spindle power to estimate feedrates minimizing the production time	Offline spindle power and feedrate (in the previous operation)	Milling
Offline	Spindle power control [14]	Multi-objective optimization is developed to improve machining efficiency and reduce fluctuations in the spindle power based on an ANN-based model of spindle power		Milling
Offline	Cutting force control [164]	A machining time minimizer is developed based on the simulation of cutting engagements and predicting cutting forces. The optimizer maximizes the cutting forces through the tool path by manipulating the feedrate		Milling
Online	Cutting force control [165]	An online force control system was developed that automatically adjusts feedrate based on the force signal. To prevent vibration damage, a chatter suppression control module was added to the system by analyzing the force feedback.	Force sensor	Turning
Online	Cutting force control [166]	Nonlinear mechanistic machining force model identification with Bayesian inference and recursive least square estimator	Directional strain gauge-based force sensors	Turning
Offline/ Online	Cutting force control [162]	Combination of offline cutting force optimization using artificial neural network (ANN) as the predictive model and particle swarm optimization (PSO) along with online feedforward force control using neural control to adjust the feedrate by assigning a feedrate override percentage	Cutting force signals	Milling
Offline/ Online	Cutting force, dynamic stability and cutting temperature [13]	A hybrid optimization, monitoring, and control (HOMC) system was introduced considering the machining primary limits of chatter, tool deflection, and thermal stresses	Spindle power, vibration and acoustic emission	Milling

In other applications, multi-objective process optimization schemes were developed in order to minimize cutting characteristics such as energy consumption [167] and burr formation [168,169], along with surface roughness and part quality. Productivity and part quality are critical metrics in manufacturing that can be defined through the combination of the mentioned objectives. These metrics are shaped by a multitude of factors, including reducing machining time, enhancing surface quality, and optimizing material removal rate (MRR) or machining force across the entire production process. However, one crucial aspect often overlooked is the impact of tool wear on achieving these objectives. Therefore, a comprehensive approach that integrates considerations of tool wear alongside these aforementioned objectives is crucial. The Pareto front optimal solution approach [170,171] and gray rational analysis [172,173] are the common approaches for defining the trade-off between objectives. These approaches can be embedded in a Techno-Economic module of a cyber–physical system for the integration of different objectives.

### 4.2. Part Quality

In this section, attention is directed towards the enhancement of part quality within the framework of cyber–physical systems. The multifaceted factors crucial for refining part characteristics in manufacturing environments are studied. Emphasis is placed on mitigating challenges such as Geometric Accuracy (Section 4.2.2), with a specific focus on tool deflection, and Surface Integrity (Section 4.2.1), entailing the management of phenomena such as chatter and residual stresses.

#### 4.2.1. Surface Integrity

Self-exited vibrations in machining processes, known as chatter, are one of the deteriorating phenomena that lead to poor surface quality, reduced tool life, reduced spindle life, and decreased productivity. The two known chatter mechanisms in machining processes are regenerative waviness and mode coupling [174,175]. In regenerative waviness, a cutting edge is engaged with a wavy surface that is already machined in the presence of periodic cutting forces. Due to the phase difference between the wave of the cutting-edge motion and the surface wave in the new engagement, the chip thickness and cutting forces vary, causing a diverging vibration condition. The mode coupling phenomenon occurs due to merging two or more sources of vibration, e.g., when vibration in the thrust force direction generates vibration in the cutting force direction and vice versa [176]. The regenerative waviness, which occurs more frequently in CNC machining, is the most-studied chatter mechanism. However, in robotic machining, which is characterized by low structure stiffness, both chatter mechanisms are important and need to be considered [176].

**Figure 11:**
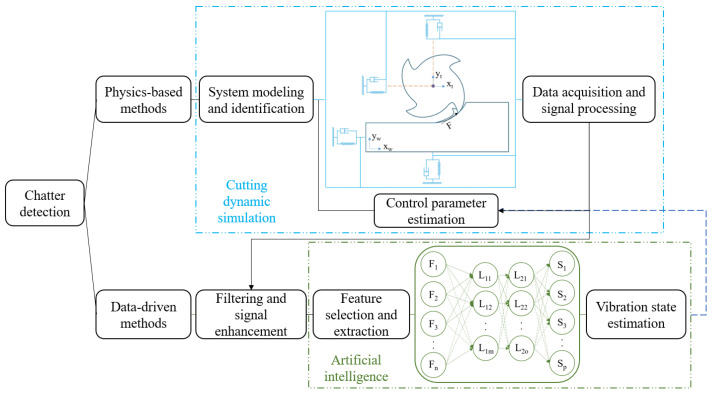
Machining chatter vibration detection and suppression strategies.

As shown in Figure 11, the main chatter detection strategies can be categorized into physics-based and data-driven methods. The physics-based chatter detection methods are more reliable and accurate in machining using a specific machine tool equipped with the chatter detection sensors and when the dynamic behavior of the system is known. The main difficulty in detecting chatter is differentiating between the stable and unstable vibration modes during a cutting process with the existence of multiple sources of vibrations with a varied range of frequencies. These changes frequently occur in cutting conditions at the beginning and end of a cutting engagement, as well as when the feed direction changes, and may result in temporarily unstable vibration conditions, to which physics-based chatter detection methods are particularly sensitive.

The second strategy to detect chatter is to develop data-driven models, where external sensors such as accelerometers, AE sensors, and dynamometers have been used to directly detect chatter in machining operations [177,178]. Signal-based data-driven chatter detection algorithms can be employed in real-time control systems to actively suppress chatter vibrations through modifying the spindle speed. These algorithms have recently been emphasized due to their capability to address highly nonlinear phenomena. To increase the feasibility of utilizing chatter stability analysis in industrial applications, a data-driven model was recently developed using deep neural networks (DNNs), with spindle speed, depth of cut, tool clamping length, entry angle, and exit angle selected as the model inputs [179]. This approach eliminates the necessity for the tool tip dynamic measurement, as well as the estimation or measurement of cutting forces. In this model, the results of an analytical simulation were used to pre-train the data-driven model. A specialized adaptive chatter suppression system was developed based on the adaptive spindle Speed Difference Method (SDM), along with an observer-based chatter state extraction for a parallel milling process [180]. This system successfully evaluated chatter frequencies during a particular machining operation and sequentially varied the spindle speed accordingly. However, these approaches require further investigation to improve their accuracy for reliable implementation in industrial environments. The control system must be able to deal with a wide range of cutting engagements and mechanisms to be used in industrial applications. A possible improvement is to combine a data-driven model with a physics-based model to enhance the generality of the approach, as recently introduced in [181]. It is important to note that spindle speed is not a suitable cutting parameter to be manipulated in real time during a machining process, as it can have negative effects on the surface quality of the machined parts, in addition to safety-related issues and concerns.

Table 7 represents a summary of the developed chatter detection methodologies for machining processes. As can be seen in the table, the dominant strategy to eliminate chatter vibration in the literature is offline optimization. The most common way to avoid chatter is to evaluate the stability lobe diagram (SLD) based on the machine–tool setup dynamic characteristics, mechanical and cutting properties of the workpiece material, and the range of the cutting conditions. The SLD defines the stable and unstable depth of cuts at each spindle speed, which can be determined using an impact test prior to the cutting operation [182]. This approach avoids the complications and uncertainties involved in the online approaches and, therefore, it is more feasible for implementation in industrial environments. An example of this approach is the offline optimization methodology developed in [183], in which SLD information was used to establish the minimum and maximum bounds of the tool life and MRR and superimposed onto the stability lobes in order to evaluate the cutting condition with the lowest cost. With the advent of data-driven approaches to detect chatter states, their implementation of online optimization processes became possible due to their capability to deal with complex cutting conditions. In addition, these approaches can be combined with a physics-based approach to provide training data to further improve their efficiency and comprehensiveness. Another observation is that chatter avoidance is usually implemented as a constraint in the multi-objective optimization of machining processes to determine the optimum cutting conditions, considering objectives such as machining time and energy consumption. This holistic approach ensures a more comprehensive and effective optimization strategy for machining operations, which contributes to a more adaptable and intelligent system that addresses diverse machining challenges.

Numerous product and process features may require optimization, depending on the manufacturers’ production priorities and constraints. The common crucial features of industrial products are the surface roughness [184,185], dimensional accuracy [186,187], cutting temperature [188,189], and machining-induced residual stresses (RS) [190]. Surface roughness is an essential quality indicator, since it influences the mechanical characteristics of the final product, such as wear, corrosion, lubrication, thermal and electrical conductivity, and fatigue behavior [191,192]. For instance, process optimization can be formulated to determine the optimum combination of cutting parameters to enhance the surface quality and dimensional accuracy while taking into account machining errors, such as tool run-out and deflection and spindle vibration [193]. In the investigation reported in [194], the optimum combination of micro-milling parameters to obtain the desired accuracy and surface roughness was determined through experimental modelling and particle swarm optimization (PSO). The same approach has been used to optimize the surface roughness and form errors in the ball end milling of free-form surfaces based on the experimental modelling of influencing parameters and gray rational analysis [195]. In a similar study, digital twin-driven surface roughness and tool wear prediction models were proposed based on Improved Particle Swarm Optimization-Generalized Regression Neural Networks (IPSO-GRNN) to adaptively control the process parameters to improve the quality and efficiency of the production [196]. In studies on optimizing the cutting parameters in the end milling of Ti6Al4V, the temperature and surface roughness of the final product were optimized based on neutral network modelling and a PSO algorithm [197]. As experimental findings showed, the surface roughness is primarily impacted by the depth of cut, whereas the cutting speed and feed rate have no significant impact during the high-speed machining of titanium alloys [198]. The effect of temperature was also investigated in the end milling process of Al 6063, considering parameters such as the helix angle, spindle speed, feedrate, and axial and radial depth of cuts [199].

As widely investigated, the effect of temperature is crucial on tool wear in machining hard-to-cut materials such as titanium alloys. In addition, the cutting temperature influences the distribution of residual stresses on the machined surfaces through phase transition and thermal expansion [200,201]. A systematic data-driven fuzzy modeling technique utilizing the Non-Dominated Sorting Genetic Algorithm II (NSGA-II) was used to find the cutting conditions that generate compressive surface stresses or minimize the tensile stresses of the machined surfaces [202]. As presented in [203], an optimization procedure based on the combination of a data-driven model, Support Vector Regression (SVR), and improved PSO was developed to determine the optimal process parameters and ensure that the tensile residual stress on the product surfaces complied with the design requirements. Process optimization can also be developed for specific material and cutting conditions such as machining carbon fiber reinforced composites (CF/PEEK) under dry cutting conditions, in which controlling the surface defects is highly crucial [188]. To address this problem, a process optimization method to improve the machining efficiency and reduce the surface defects was developed for the high-speed dry milling of CF/PEEK material, based on an analysis of the thermal impact of the cutting process on the machined surfaces [188].

**Table 7:** Developed machining process optimization methodology based on chatter avoidance.

Approach	Objective	Methods	Feedback	Machining Process
Offline	Chatter Avoidance [204]	A heuristic approach is developed to determine the range of spindle speed from the stability lobe diagram to be used in minimization of energy consumption and machining time by selecting the optimum feedrate, depth, and width of cut		Milling
Offline	Chatter Avoidance [205]	A multi-objective optimization methodology to maximize MRR and minimize surface location error (SLE), considering a_plim_ as the depth constraint to avoid chatter vibration		Milling
Offline	Chatter Avoidance [206]	Using the determined relationship between the lead angle and depth of cut from an experimentally constructed chatter stability lobe diagram, an iso-planar tool path is generated to maximize the depth of cut in a five-axis milling operation		Five-axis milling
Offline	Chatter Avoidance [207]	A chatter-free machining approach is developed to maximize the allowable cutting depth based on genetic algorithms. The method optimizes several tool parameters such as number of teeth, shank diameter, fluted section diameter, shank length, taper length, and length of fluted section		Milling
Online	Chatter Avoidance [208]	Constructing the transfer function of a spindle velocity controller by measuring the Frequency Response Function (FRF) of the system	Drive motor current signals	Milling
Online	Chatter Avoidance [180]	Adaptive spindle speed difference method (SDM)	Sensor-less cutting force estimation	Parallel end-milling

#### 4.2.2. Geometric Accuracy

Tool deflection estimation is a critical aspect in machining applications, influencing the precision of the geometric accuracy of the manufactured components. It is highly influenced by the geometrical variations of the product, as well as the cutting parameters. The determination and control of tool/workpiece deflections are crucial, especially when the compliance of the tool or workpiece is high. High-speed rough-end milling [209] and the machining of thin-wall workpieces [210] are examples for cases in which the deflections of the tool and workpiece are crucial for obtaining the desired geometric accuracy of the final part while maintaining a high productivity. It is usually constrained to avoid a sudden tool failure, the deterioration of the surface integrity of machined part, or part scrapping. Traditionally, experimental approaches were adopted to find the optimal cutting parameters to avoid the inaccuracies caused by tool and workpiece deflections. These approaches are not economically feasible for medium and low production rates.

Various models and implementations have been developed to address this challenge and enhance machining processes. The offline estimation of tool/workpiece deflections during a machining process include analytical approaches, where the tool can be considered as a cantilever beam, or a finite element simulation of the cutting tool and workpiece, and the evolution of the contact points between the cutting tool and workpiece [211]. FEA is commonly employed to simulate the complex interactions between the cutting tool and workpiece, providing insights into the deformation and deflection of the tool. These methods are mainly implemented at the preprocessing stage of the tool path generation and leverage advanced computational algorithms, taking into account factors such as cutting forces, material properties, and tool geometries. Additionally, machine learning techniques have been increasingly utilized to predict tool deflection based on historical data and real-time sensor inputs [212,213]. It is worth noting that CNC machines operate using specific sets of codes known as G-codes, which convey machining parameters. These optimization procedures can be applied to G-codes generated by Computer-Aided Manufacturing (CAM) software, as presented in [214,215].

By accurately estimating tool deflection, manufacturers can optimize cutting parameters, reduce tool wear, and improve the overall efficiency and precision of machining operations. The process optimization scheme tries to keep the cutting force below the maximum allowable cutting force, which produces acceptable tool or workpiece deflections. Therefore, it is important to define a tool deflection constraint during the optimization of machining processes in terms of MRR. Table 8 presents a summary of the developed optimization systems considering tool/workpiece deflection. As can be seen offline, the minimization or compensation of the tool and/or workpiece deflections is conducted to optimize the cutting process in terms of MRR within the constraints of tool wear and surface roughness. Each of these approaches is suitable for different applications. The minimization of the deflections is important in the machining of thin-wall structures, while its compensation is more significant in roughing and semi-finishing operations. Imposing surface roughness as a constraint by reducing the deflection is critical in finishing operations. The majority of these methods were developed based on the offline optimization approach, due to the negligible uncertainty in the deflection prediction compared to other phenomena, such as tool wear and chatter vibration. This is particularly true when 3D FEA is used, since computation time is not a limiting factor.

### 4.3. Process Sustainability

From the energy consumption point of view, manufacturing accounts for the largest share of annual industrial energy consumption, consuming about 54% of the world’s total delivered energy [221], demonstrating the critical impact of manufacturing operations on the environment [222]. Traditionally, machining energy is determined by estimating the cutting forces to remove a specific amount of material, which is known as the energy consumption of the tool-tip [223]. However, other sources of energy consumption, such as axillary equipment consumption and waste generated during production, must be accounted for in the estimation of consumed energy [224]. In addition, it is important to consider various qualitative factors, such as the operator’s health, the shop floor environment, air quality, and the environmental impact of coolant/lubricant to achieve sustainable production [225]. It has been demonstrated that around 66% of the total consumed energy is used for the actual cutting process, indicating the importance of optimizing the cutting processes in terms of energy consumption [226]. Historically characterized by a substantial energy demand, machining processes have been associated with notable environmental concerns. Efforts to curtail the industry’s ecological footprint are actively underway, with a focus on technological advancements and strategic interventions designed to mitigate energy consumption. The emerging I5.0 paradigm shift towards integrated sustainability and high-performance machining was analyzed by Attia [6], who emphasized the need to continuously seek various avenues to increase productivity, reduce cost, and reduce energy consumption through process optimization, the development of hybrid new processes, and adaptive control strategies.

The two primary branches of studies on manufacturing energy consumption are studies on designing machines and equipment to reduce the spent energy [227] and investigations on the optimization of cutting parameters [228]. The main elements influencing the energy consumption in a machining operation are the cutting parameters, such as feedrate, spindle speed, depth of cut, and cutting fluid settings. It has been experimentally found that feedrate has the highest effect collectively on energy consumption, surface roughness, and vibration [229]. Cutting tool selection is also an essential factor in process optimization, since it determines the range of cutting parameters [144,230]. The optimization of cutting parameters, incorporation of eco-friendly lubricants, and exploration of energy-efficient machining technologies are building blocks of the I5.0 paradigm shift.

**Figure 12:**
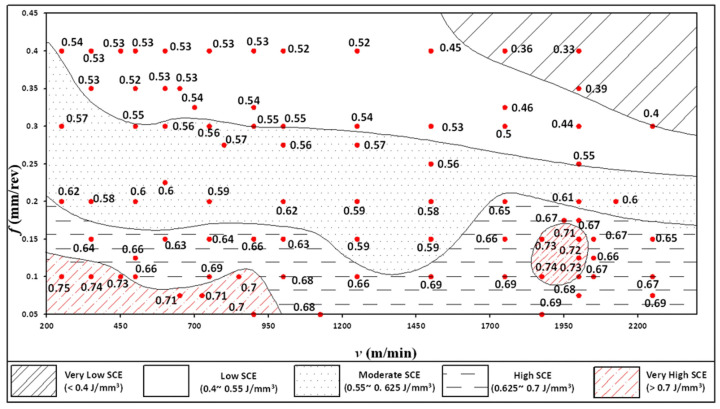
Energy consumption map generated for machining Al 6061-T6 [233].

The first step in optimizing energy consumption is to develop a model correlating the cutting inputs with the consumed energy. Analytical [222], empirical [231], and data-driven [232] models have been developed to estimate the objective function. These models can be further combined with other objectives such as machining time, part quality, and tool wear to improve the overall performance of the process. It has been found that energy consumption and carbon emission are greatly affected by the tool wear state, which reveals the importance of combining multiple objectives to acquire a higher efficiency [129]. Energy consumption maps have been generated for a variety of machine–tool–material combinations, using the same approach presented for the tool wear [141,233]. Figure 12 shows an energy consumption map developed for machining Al 6061-T6 using an uncoated tool. The map identifies the cutting conditions that result in the lowest specific cutting energy (SCE) index. The SCE index is defined as the cutting power fraction divided by the MRR (J/mm^3^). Additionally, sustainability in machining extends beyond energy considerations, encompassing waste minimization, resource optimization, and the integration of circular economy principles to develop a more ecologically responsible machining ecosystem. A paradigm shift is evident in the industry’s commitment to low-carbon machining technologies, adoption of renewable energy sources, and investigation into carbon capture and storage methods, all aimed at limiting carbon emissions.

Table 9 presents the developed optimization systems to reduce energy consumption and carbon emission in machining processes. As can be seen in the table, the offline approach is the main strategy to optimize the machining processes in terms of these objectives. However, the variations in cutting conditions during a machining process, which affects objectives such as tool wear rate, cutting temperature, and chatter vibration, could have a significant effect on the machining energy consumption. The important trade-off in machining process optimization is between product quality and the consumed energy, which commonly have an inverse relationship. Thus, it has been recommended to consider product quality, specifically surface roughness, as a constraint in the optimization process rather than an objective [234]. It can be concluded that optimizing the cutting parameters is not sufficient, on its own, to reduce energy consumption; monitoring cutting conditions to avoid deteriorating events is also essential in achieving this goal.

## 5. Gap Analysis and Future Outlook

Section 3 discussed the virtual machining systems that consisted of various models, including FEMs of the cutting operation and tool crack propagation, tool/workpiece deflection, surface roughness estimation, engagement geometry, and machine–tool–workpiece dynamic models. In Section 4, the main objectives of process optimization and examples of their implementation were reviewed. The effectiveness of these methods depends on the accuracy and comprehensiveness of the cutting state predictions.

**Figure 13:**
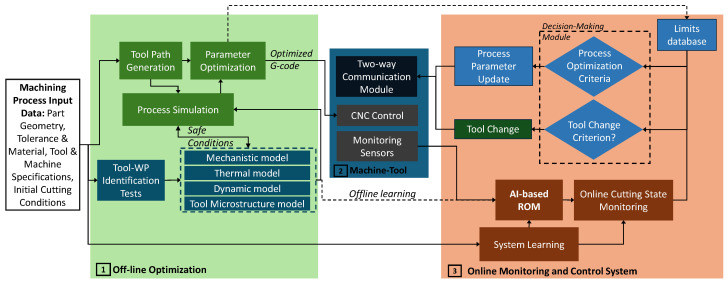
Envisioned cyber–physical system for high-performance machining.

While there have been some studies that have attempted to combine multiple models and optimization techniques for both online and offline strategies, a comprehensive optimization approach is still lacking. This approach should consider the appropriate models to be implemented in offline and online optimization modules and be able to handle the complexity, nonlinearity, and unknown external disturbances of the machining process. Additionally, to create a reliable industrial intelligence system, dynamic data collected from process sensors should be linked with advanced computer modelling and simulation. Significant progress has been made in developing reliable and quick communication systems for machining setup and introducing advanced models that integrate AI with analytical and numerical models. These advances provide the opportunity to develop a cognitive cyber–physical system for machining platforms. Virtual models with low computation efforts to estimate the static, dynamic, and thermal states of a cutting process need to be constructed and swiftly integrated in real-time adaptive control CPSs. This is to achieve the terminal objectives of reducing errors and uncertainties while improving process time and cost. Therefore, such a CPS should consist of a suite of offline simulations and optimization and adaptive real-time control system. Offline analytical and numerical models to predict the cutting states and model-based process optimization approaches need to be developed to predict the safe cutting conditions, considering process dynamic stability [24,181], tool wear [117,140], and machined part geometric accuracy and surface integrity [45,187]. This should be integrated with online optimization modules that are developed to adaptively control the feedrate during the machining process, considering the uncertainties during the cutting operations. Based on the conclusions drawn from this thorough analysis of the available literature, an ideal CPS for a high-performance machining system is envisioned, as shown in Figure 13.

A cyber–physical system for high-performance machining should consist of both offline and online optimization modules. The offline optimization module should include mechanistic [64,89], thermal [61], dynamic [182,213], and tool microstructure models [103,109]. These high-fidelity models predict the cutting states and optimize the thermal and mechanical loads on the tool along the tool path. To integrate these models in real time for digital twinning and overcome their high computational and time needs, AI-based reduced order modeling (ROM) is a potential candidate. ROM, augmented by AI, encompasses a suite of automated computational techniques devised to facilitate the repurposing of intricate models for the generation of swifter, less detailed approximations. This enables integrating physics-informed AI models in real time to further improve the generality and accuracy of the cutting state predictions. The models can be further integrated to construct a digital twin for the machine–tool and process. These AI-ROM models can be utilized in an online monitoring and control system, where the cutting states are determined through monitoring sensors. Process-born feedback signals are integrated with AI-ROM models to provide instantaneous predictions of different process states with a comparable accuracy to high-fidelity time-consuming FEA models. These predictions can be utilized by a decision-making module to take corrective actions in real time to achieve the objectives of the CPS. However, as shown in the conducted literature review, there is a need for further investigation into AI-driven FEA and ROM models to enhance the efficiency of analysis computations and enable real-time microstructure FEA with a high number of cycles, which reflects the relevant range of the industrial process.

An essential component of the online optimization process is the decision-making module that controls the cutting parameters based on various criteria. Within the safe cutting conditions defined offline, this module can take corrective actions by updating the process parameters to increase the process productivity while maintaining the surface integrity of the machine part. Additionally, an important criterion is the tool change, which can be separately defined to interrupt the cutting process to avoid tool failure. To achieve this multi-aspect CPS system, the following are the future outlines that have been drawn from the current state of the art:

- Establishing a connection between cutting state numerical models and empirical and AI-based ones to improve their accuracy and reduce the time and cost of the experimental procedure performed to develop them. This is needed as a result of the technical sophistications required for the implementation of numerical models in industrial applications.
- Conducting further research studies to optimize cutting parameters that are directly linked to process sustainability. These optimization approaches have recently become in high demand due to the emergence of new aspects that must be considered in industrial production driven by the emerging regulatory obligations and policies related to climate action and energy consumption.
- Performing further studies to investigate crack propagation that can be used to correlate the propagation characteristics with the machining signals, such as AE for the early prediction and prevention of tool failure.
- Combining offline machining system models with online monitoring and multi-objective optimization approaches to provide an all-inclusive cyber–physical machining system that maximizes manufacturing productivity and improves process sustainability and profitability.

## 6. Conclusions

In this comprehensive study of process optimization for cyber–physical systems’ development, key aspects critical for enhancing machining efficiency were investigated. Section 1 provided an overview of the process optimization strategies and the evolution of cyber–physical systems. Section 2 presented the framework of cyber–physical systems for machining processes and their main components. In Section 3, the material constitutive models, fracture models in chip formation, thermal boundary conditions, heat transfer models, and microstructure modelling were discussed and benchmarked for cyber–physical system development. These discussions addressed the challenges facing Industry 5.0 and emphasized the role played by numerical models in achieving comprehensive modelling. The diverse objectives and constraints associated with process optimization though cyber–physical systems were explored in Section 4, encompassing offline, online, and hybrid approaches. Additionally, the cutting-edge methodologies for process optimization, with dedicated attention to productivity and economics, part quality, and process sustainability, were discussed. The different aspects of process optimization methodologies have been explored to recognize their capabilities, limitations, and opportunities to be implemented for industrial applications.

Finally, a gap analysis was conducted to highlight the research gaps in state-of-the-art CPS-based machining process optimization. This in-depth analysis of the available literature showed that the recent advances in signal processing and data-driven modelling techniques provide a suitable platform for cyber–physical machine tool system development. More efforts should be directed toward AI-based reduced order modelling to facilitate the implementation of high-fidelity numerical models in real-time process monitoring and control. Based on the explored advancements in both the software and hardware aspects of machining systems, an envisioned cyber–physical system for machining has been introduced. It is worth mentioning that the implementation of the developed optimization methods for industrial applications is still limited due to the lack of comprehensiveness and autonomy for decision making in the developed methods. In terms of virtual modelling, the implementation of an accurate FE model to simulate complex phenomena such as chip formation and predict tool chipping and tool wear is highly effective in improving the accuracy of the optimization process. Other numerical methods, e.g., molecular dynamic simulation (MDS) and Smoothed-particle Hydrodynamics (SPH), need to be further investigated for the current application that involves the machining simulation of conventional hard materials and nano-crystalline materials at the atomic- and nano-scales.

## Figures and Tables

**Table 1:** Identified Johnson–Cook parameters for Ti6Al4V reported in the literature.

Model	*A* (MPa)	*B* (MPa)	*n*	*m*	*C*	${\dot{\varepsilon }}_{0}$(1/s)
JC-1 [79]	782.7	498.4	0.28	1	0.028	10^−5^
JC-2 [80]	896.4	649.5	0.387	0.758	0.0093	1
JC-3 [81]	870	990	1.01	1.4	0.008	1
JC-4 [82]	1098	1092	0.93	1.1	0.014	1

**Table 2:** Modified Zerilli–Armstrong model constants for Ti6Al4V [80].

$C_{1}$	$C_{2}$	$C_{3}$	$C_{4}$	$C_{5}$	$C_{6}$	*n*	$T_{ref}$
869.4	640.50	0.0013	−9.57 × 10^−4^	0.0095	6.94 × 10^−6^	0.3867	323

**Table 3:** Voyiadjis–Abed model constants for Ti6Al4V [84].

$C_{1}$	$C_{2}$	$C_{3}$	$C_{4}$	$C_{5}$	$C_{6}$	$C_{7}$	${\dot{\varepsilon }}_{p}^{*Y}$	${\dot{\varepsilon }}_{p}^{*H}$
30	500	0.11	1400	4.2 × 10^−5^	1100	0.5	1.16 × 10^13^	2.6 × 10^13^

**Table 4:** Heat transfer boundary conditions in machining.

Cooling Method	Initial Temperature (°C)	Heat Transfer Coefficient (Wm^−2^ K^−1^)
Dry cutting [86]	20	10–20
High-pressure coolant (HPC)	20	20 × 10^3^–55 × 10^3^
Minimum Quantity Lubrication (MQL) [100,101]	20	200–3 × 10^3^
Cryogenic machining [95,101]	20	30 × 10^3^–50 × 10^3^

**Table 5:** Developed machining process optimization for tool wear.

Approach	Objective	Methods	Feedback	Machining Process
Offline	Tool wear [149]	An experimental approach using RSM is developed to identify the most significant cutting parameters on surface roughness, flank wear, and acceleration of drill vibration velocity. The optimal parameters are determined using a multi-response optimization algorithm	Acousto-Optic Emission (AOE) signal (laser Doppler vibrometer)	Drilling
Online	Tool wear [144]	A multi-objective optimization of flank tool wear, cutting forces, and machining vibrations is developed using an experimental RSM-based approach	Cutting forces and vibrations	Turning
Offline	Tool wear [150]	An experimental procedure is conducted to minimize the flank wear and crater using regression modelling, desirability analysis, and GA algorithms in the machining of Al alloy and SiC composites	-	Turning
Offline/Online	Tool wear control [21]	Taguchi experimental design and optimization are used to minimize flank wear in the machining of AISI 1050 material, considering cutting speed, feed rate, and tool tip type as the inputs	Tangential cutting force and AE signals	Turning
Offline/Online	Tool wear control [151]	Model-based force-wear predictor along with delamination and/or thermal damage estimator [152]—stepwise decision making	Motor power signal	Drilling
Offline/Online	Tool wear control [129]	Multi-objective optimization to minimize tool wear and surface roughness and maximize MRR is developed based on an adaptive neuro-fuzzy inference system (ANFIS) for modelling and the vibration and communication particle swarm optimization (VCPSO) algorithm for the optimization	Cutting forces	Milling

**Table 8:** Developed machining process optimization for tool deflection.

Approach	Objective	Methods	Feedback	Machining Process
Offline	Tool deflection minimization [216]	A methodology was developed to reduce deflection errors in end milling. Parameters such as lubrication mode (flood, MQL, nano lubrication, dry), axial depth of cut, radial depth of cut, and feed rate were studied experimentally using the Taguchi method. The results showed that the cutting forces and the distance between the tool holder and workpiece have the greatest impact on deflection errors	-	Milling
Offline	Workpiece deflection constrained [217]	A methodology to maximize MRR is developed considering a penalty cost function of the deflections that occur during thin-wall machining. Radial depth of cut, axial depth of cut, spindle speed, feed per tooth, and number of flutes are considered as the input parameters	-	Milling
Offline	Tool and workpiece deflection [218]	An experimental design using RSM is conducted to minimize the tool and part deflection in the machining of a thin-wall workpiece considering feedrate, spindle speed, and depth of cut as the cutting parameters	-	Milling
Offline	Tool deflection [219]	Finite element modeling of the cutting tool and workpiece based on a mechanistic approach to determine cutting forces	-	Milling
Online	Tool deflection compensation [220]	A method is developed that utilizes the drive signals to compensate for tool deflections. Based on the evaluated forces from the machine tool’s drive signals, the tool path is compensated orthogonal to the feed direction	Drive signal	Milling

**Table 9:** Developed machining process optimization for energy consumption and carbon emission.

Approach	Objective	Methods	Feedback	Machining Process
Offline	Energy consumption [226]	Multi-objective optimization of cutting parameters to reduce energy consumption and increase production rate in the milling operation of aluminum alloys	-	Milling
Offline	Energy consumption [235]	Minimization of cutting specific energy consumption and processing time by considering surface roughness, maximum power, and tool life as constraints using a quantum genetic algorithm	-	Milling
Offline	Carbon emission [27]	Cutting time, machining cost, and carbon emission are minimized using non-cooperative game theory integrated with NSGA-II. Tool path and cutting parameters (feed per tooth, spindle speed, and depth of cut) are optimized based on the developed model and an improved GA algorithm	-	Milling and turning
Offline	Carbon emission [234]	To minimize carbon emission and machining time, an optimization process is developed based on statistical modelling of process responses considering surface roughness as a constraint and cutting speed, feedrate, and depth of cut as the optimization parameters	-	Turning
Offline	Energy consumption [236]	The energy consumption and manufacturing time are minimized through a multi-objective optimization of machining center process routes using work step chain intelligent generation algorithm and NSGA-II	-	Milling, boring, and drilling
Offline	Carbon emission [237]	The optimal cutting parameters and the cutting tool have been selected through a multi-objective optimization of machining carbon emission, time, and cost using the NSGA-II algorithm	-	Turning
